# Modifications of 24-h movement behaviors to prevent obesity in retirement: a natural experiment using compositional data analysis

**DOI:** 10.1038/s41366-023-01326-0

**Published:** 2023-05-23

**Authors:** Kristin Suorsa, Nidhi Gupta, Tuija Leskinen, Lars L. Andersen, Jesse Pasanen, Pasan Hettiarachchi, Peter J. Johansson, Jaana Pentti, Jussi Vahtera, Sari Stenholm

**Affiliations:** 1 Department of Public Health, University of Turku and Turku University Hospital, Turku, Finland; 2 Centre for Population Health Research, University of Turku and Turku University Hospital, Turku, Finland; 3 National Research Centre for the Working Environment, Copenhagen, Denmark; 4 Department of Medical Sciences, Occupational and Environmental Medicine, Uppsala University, Uppsala University Hospital, Uppsala, Sweden; 5 Clinicum, Faculty of Medicine, University of Helsinki, Helsinki, Finland

**Keywords:** Risk factors, Epidemiology

## Abstract

**Background:**

Retirement often leads to a more passive lifestyle and may therefore lead to weight gain. This study aims to investigate longitudinal associations between changes in 24-h movement behaviors and BMI and waist circumference in relation to the transition from work to retirement.

**Methods:**

The study population included 213 retiring public sector workers (mean age 63.5 years, standard deviation 1.1) from the Finnish Retirement and Aging study. Before and after retirement participants wore an Axivity accelerometer on their thigh and filled in a daily log for at least four days to measure daily time spent sleeping, in sedentary behavior (SED), light physical activity (LPA) and moderate-to-vigorous physical activity (MVPA). Also, their body mass index (BMI) and waist circumference were measured repeatedly. Compositional linear regression analysis and isotemporal substitution analysis were used to study associations between one-year changes in 24-h movement behaviors and concurrent changes in BMI and waist circumference.

**Results:**

An increase in MVPA in relation to sleep, SED and LPA was associated with a decreasing BMI (β = −0.60, *p* = 0.04) and waist circumference (β = −2.14, *p* = 0.05) over one year from before retirement to after retirement. In contrast, increasing sleep in relation to SED, LPA and MVPA was associated with an increasing BMI (β = 1.34, *p* = 0.02). Reallocating 60 min from MVPA to SED or sleep was estimated to increase BMI by on average 0.8–0.9 kg/m^2^ and waist circumference by 3.0 cm during one year.

**Conclusions:**

During the transition from work to retirement, increasing MVPA was associated with a slight decrease in BMI and waist circumference, whereas increasing sleep was associated with an increasing BMI. Common life transitions, like retirement, should be considered when giving recommendations and guidance for physical activity and sleep.

## Introduction

Obesity has grown to epidemic proportions worldwide and causes major disease burden including higher risk of cardiovascular diseases, type 2 diabetes and mental health problems [1–3]. Sufficient moderate-to-vigorous physical activity (MVPA) is a key factor in preventing obesity [4]. However, time spent in MVPA forms only a small proportion of a 24-h day and the remaining time is spent in other activities, often referred as 24-h movement behaviors, i.e., sleeping, sedentary behavior (SED) and light physical activity (LPA), which all contribute to daily energy expenditure. Researchers have traditionally studied 24-h movement behaviors in isolation from each other and results suggest that not only MVPA, but also LPA is beneficially associated with obesity indicators [5], while high SED is associated with higher risk of obesity [6]. Moreover, short sleep has been consistently associated with obesity [7]. Some indications on association between long sleep and obesity have also been reported [8], pointing out to *U*-shaped association that is often seen with respect to sleep and mortality [7, 9]. The main limitation in these previous studies is that they have not taken into account the codependency between the behaviors. Movement behaviors are bound to a 24-h day, meaning that increasing time in one behavior inevitably decreases time used in at least one of the remaining behaviors. Recently there has been a shift towards the 24-h time use paradigm, that is, examining all behaviors as relative components of a 24-h day [10–12].

Growing evidence suggests that the way we allocate our time between various movement behaviors throughout the day can have a significant impact on our overall health. Therefore, it’s important to consider the 24-h time use paradigm when analyzing these behaviors [13]. For instance, reallocating time to MVPA may result in different changes in obesity indicators depending on whether time is allocated from sleep or SED. This is because sleep is known to be important for glucose metabolism and hormone levels regulating hunger and appetite [14], while SED is considered as waking behavior characterized by low energy expenditure. While a plausible recommendation in obesity prevention would be to reallocate time from SED but not from sleep to MVPA, evidence based on the 24-h time use paradigm is still lacking.

Compositional data analysis (CoDA) is a fairly new and feasible approach to examine relative changes in 24-h movement behaviors and to study health effects of time reallocations between the behaviors [11, 12]. Previous studies applying CoDA show lower BMI, fat mass and waist circumference in adults who spend more time in MVPA in relation to the remaining behaviors [12, 15–23]. Moreover, spending more time in LPA in relation to SED/sleep as well as in sleep in relation to SED have been suggested to be associated with lower BMI and waist circumference [12, 19–21]. However, the main limitation in these previous CoDA-based studies is that they are cross-sectional and thus unable to examine how within-individual changes in 24-h movement behaviors are associated with concurrent changes in obesity indicators. Only one previous longitudinal study on this topic exists, indicating reallocation of time from SED to MVPA to be associated with decreases in BMI and adiposity (and vice versa) over seven years among older adults [23]. However, this study did not include sleep, an important component of a 24-h day. Thus, it is unknown how within-individual changes of all 24-h movement behaviors, including sleep, are associated with changes in obesity indicators.

The transition to retirement offers a natural experiment setting to examine how changes in 24-h movement behaviors are associated with changes in obesity indicators. Removal of work hours and increased leisure time modifies time use and may consequently change 24-h movement behaviors. Previous studies have shown that the transition from work to retirement is associated with an increased sleep duration [24], decreased physical activity, and increased sedentary time among manual workers [25–27], and slightly increased physical activity among non-manual workers [25]. We have recently applied the CoDA methodology to examine changes in 24-h movement behaviors during the retirement transition and found that the proportion of sleep increases in relation to physical activity at the population level [28]. The proportion of SED also changes, with increases observed among manual workers and decreases observed among non-manual workers [28]. The transition to retirement has also been linked to increased BMI [29, 30], but it is unknown whether changes in 24-h movement behaviors are associated with changes in BMI or other obesity indicators.

To fill the research gaps, we conducted repeated annual accelerometer and clinical measurements in aging employees over their transition from work to retirement. The aim of this study was to examine how within-individual changes in the composition of 24-h movement behaviors are associated with changes in BMI and waist circumference over one year by applying the CoDA methodology.

## Methods

### Study design and participants

The study population consisted of participants from the Finnish Retirement and Aging Study (FIREA), an ongoing longitudinal cohort study of older adults in Finland established in 2013. Details of the design and implementation of the FIREA study have been reported elsewhere [31]. Shortly, participants were first contacted 18 months prior to their estimated retirement date by sending them a questionnaire. After responding to the questionnaire, Finnish-speaking participants with estimated retirement date between 2017 and 2019, who lived in Southwest Finland and were still working, were invited to participate in the clinical sub-study (*n* = 773). Of them, 290 agree to participate. Thereafter study participants have been followed up with annual measurements including questionnaires, clinical and accelerometer measurements. To determine the timing of retirement, the actual retirement day was inquired during each phase of the data collection, and this information was used to determine pre- and post-retirement measurements.

Flow chart for the selection of the analytical sample is presented in Supplement 1. Of the clinical sub-study participants, 240 participants took part in simultaneous clinical and accelerometer measurements before and after the transition to full-time statutory retirement, with on average one year in between the measurements. We excluded participants who had less than three valid measurement days before and/or after retirement (*n* = 27), leaving 213 participants to the analytical sample.

### Assessment of 24-h movement behaviors

A triaxial accelerometer Axivity AX3 (Axivity Ltd Newcastle, UK) accompanied with a daily log was used to estimate 24-h movement behaviors, that is, sleep, SED, LPA and MVPA before and after retirement. Detailed description of the measurement protocol is reported elsewere [32]. During the clinical study visit a study nurse fastened the accelerometer with adhesive waterproof film dressing to the skin on the right thigh, into a standardized position [33]. Before retirement participants were asked to wear the accelerometer at least four days and nights, including at least two workdays and two days off and after retirement at least four days and nights. Moreover, participants were instructed to wear the accelerometers at all times 24 h/day, including water-based activities such as swimming, but to remove them for sauna bathing. Participants were also asked to record date, waking time, bedtime, reference measurement times and information about workday on a daily log for each day that they wore the devices.

Data from the accelerometers were downloaded through Open Movement software (version 1.0.0.37; Open Movement, Newcastle University, UK). The raw data were further processed and analyzed using a customized MATLAB program, ActiPASS (version 0.80) [34], an automatized version of Acti4 [33, 35], which determines the type and duration of different activities and body postures with a high sensitivity and specificity [33, 35]. The detailed data analysis procedures in the ActiPASS software are described elsewhere [33, 34]. We restricted the measurement period to days between the first and last date and time recorded in the daily log. Non-wear time was detected using algorithm in the ActiPASS software (≥60 min periods without movement) [33]. The measurement day was determined from midnight to midnight and a valid measurement day was defined as a day with at least 10 h of wear time during waking hours and daily log-determined waking and bedtimes. Sleep was estimated based on the bedtimes and waking times recorded in the daily log. The rest of the 24-h movement behaviors (SED, LPA and MVPA) were estimated based on the types and durations of different activities determined by the ActiPASS software. Sitting and lying time were merged into SED. Standing, moving and slow walking, with a cadence less than 100 steps/min were merged into LPA. Finally, walking fast with a cadence 100 steps/min or more [36], stair walking, running, cycling and other physical activity were merged into MVPA. All 24-h movement behavior components were averaged across all valid days.

### Assessment of BMI and waist circumference

During the two clinical visits, a study nurse measured participants’ height and weight with a wall-mounted stadiometer and a bioimbedance scale (Inbody 720, Biospace Co., Seoul, Korea) with the participants wearing only light clothing. BMI was calculated as weight in kg/(height in m)^2^. Waist circumference was measured to the nearest 0.1 cm directly on the participant’s skin, in the midpoint of the lowest rib and the iliac crest, during light exhalation in upright position. The measurement was repeated twice, and the mean value was used in the analysis.

### Assessment of pre-retirement characteristics

Sex, date of birth, and pre-retirement occupational title were obtained from the Keva Public Sector Pensions register. Participants were divided into two occupational status groups according to the occupational titles of the last known occupation preceding retirement by using the International Standard Classification of Occupations (ISCO): [37] manual workers (e.g. cleaners, maintenance workers; ISCO classes 5–9) and non-manual workers (e.g. teachers, physicians, registered nurses, technicians; ISCO classes 1–4).

Other health-related characteristics were obtained from the questionnaire preceding the transition to retirement: smoking (no/yes), self-reported doctor-diagnosed chronic diseases (angina pectoris, myocardial infarction, cerebrovascular disease, claudication, osteoarthritis, osteoporosis, sciatica, fibromyalgia, rheumatoid arthritis, and diabetes) (no/yes, one or more), mobility limitations as difficulties in walking 2.0 kilometers (no/yes) [38, 39], sleep duration (hours per night) [40], sitting time (sum of daily hours spent sitting at work, watching television, using computer at home, sitting in a vehicle and other sitting) [31] and non-occupational physical activity as metabolic equivalents (MET) hours per week [4].

### Statistical analyses

Descriptive information on participant characteristics is presented using means and standard deviations for continuous variables and frequencies and percentages for categorical variables. To examine selection to the current study, the pre-retirement participant characteristics were compared between the current study population (*n* = 213) and survey-only study population (*n* = 3698) using Chi squared test for categorical variables and ANOVA for continuous variables.

In the statistical analysis the proportion of time spent in each behavior was treated as compositional data. The analyses were conducted in the statistical software RStudio (version 4.0.5). The data set did not include zero values for 24-h movement behaviors, thus no imputation was needed.

#### Compositional data analysis

The compositional means were calculated to describe the average 24-h movement behaviors. To illustrate the heterogeneity in changes in 24-h movement behavior composition and changes BMI and waist circumference over one year, the compositional differences between pre- and post- retirement compositions were calculated for each participant [28, 41]. The compositional differences were shown by BMI and waist circumference change groups: (1) no change (<0.5 kg/m^2^ change in BMI and <3.0 cm change in waist circumference), (2) decrease (≥0.5 kg/m^2^ decrease in BMI or ≥3.0 cm decrease in waist circumference), (3) increase (≥0.5 kg/m^2^ increase in BMI or ≥3.0 cm increase in waist circumference). These cutpoints were chosen based on the distribution of the data (lower and upper quartiles) and effect sizes reported in a previous meta-analysis [42] and randomized controlled trials evaluating effects of one-year exercise programs [43–45]. The resulting composition of the compositional differences by BMI and waist circumference change groups was visualized as ternary plots.

An isometric logratio (ilr) transformation was used to map the compositional data into real-valued coordinates [46]. We used *pivot coordinates*, a specific type of ilr coordinates. The first coordinate in a set of pivot coordinates can be used to compare the proportion of one part of the composition (for instance sleep) relative to the remaining parts of the composition (that is, SED, LPA and MVPA). We created four sets of pivot coordinates to enable each behavior to be considered relative to the remaining behaviors.

We used linear regression model to examine associations between changes in 24-h movement behaviors and changes in BMI and waist circumference during the transition to retirement. The model included change in obesity indicator as the outcome variable, and changes in 24-h movement behaviors (expressed as ilrs) as the explanatory variables. Covariates included before retirement 24-h movement behavior composition (expressed as ilrs) and BMI/waist circumference, as well as age, sex and occupational status. The model was separately fitted for each set of pivot coordinates. The associations were presented as beta coefficients and their 95% confidence intervals (CI).

To illustrate the effect of observed reallocations between 24-h movement behaviors on BMI and waist circumference, we used the compositional isotemporal substitution model [47, 48]. The previously described linear regression model was used for this purpose. Systematic reallocations between movement behaviors were calculated based on the mean composition before retirement. The sizes of the reallocations were chosen to reflect the actual range of changes in the composition. Consequently, one-to-one reallocations between MVPA and one of the remaining behaviors (sleep/SED/LPA) were up to 60 min in size, while one-to-one reallocations between sleep, SED and LPA the size of one-to-one reallocations were up to 120 min in size. The regression-based coefficients were applied on the calculated difference in pivot coordinates to predict changes in BMI and waist circumference corresponding to changes in composition of movement behaviors during transition from work to retirement. The results are shown as estimated changes in BMI/waist circumference and their 95% confidence intervals (CI).

The CoDA principles applied to the current study are described in detail in Supplement 2.

As a sensitivity analysis we examined the possible seasonal effects on the associations between changes in movement behaviors and changes in BMI and waist circumference. This was done by additionally adjusting for before retirement measurement season (winter/spring/summer/autumn) and follow-up time in days in the linear regression models. Furthermore, given that associations between changes in 24-h movement behaviors and BMI and waist circumference may differ depending on whether sleep is increased from insufficient level or sufficient level [7, 8], we conducted a sensitivity analysis by excluding those reporting sleeping more than 9 h per night before retirement (*n* = 26, 12%) from the linear regression models.

## Results

Characteristics of the study population are presented in Table 1. Majority of the study population were women (82%) and non-manual workers (69%). Before retirement, the mean BMI was 26.3 kg/m^2^ (SD 4.8) and the mean waist circumference was 91.4 cm (SD 13.0) (women 89.5 cm (SD 12.8); men 100.0 cm (SD 10.7)). Participants spent on average 8.3 h sleeping, 9.7 h sedentary, 4.7 h in LPA and 77 min in MVPA per day before retirement.

**Table 1:** Characteristics of the study population (*n* = 213) before and after retirement.

Characteristics	Before retirement	After retirement
Age, mean (SD)	63.5 (1.1)	64.6 (1.2)
Women, *n* (%)	175 (82)	
Occupational group, *n* (%)		
Manual	67 (31)	
Non-manual	146 (69)	
Body Mass Index, mean (SD), kg/m^2^	26.3 (4.8)	26.2 (4.8)
Body Mass Index, *n* (%)		
Normal weight (<25 kg/m^2^)^a^	85 (40)	87 (41)
Overweight (25–29.9 kg/m^2^)	92 (43)	93 (44)
Obese (≥30 kg/m^2^)	36 (17)	33 (15)
Waist circumference, mean (SD), cm	91.4 (13.0)	90.5 (13.3)
Number of valid measurement days (range)	4.6 (3 − 10)	4.6 (3 − 7)
Number of daily log-determined nights (range)	3.1 (1 − 8)	3.2 (2 − 6)
Wear time during waking hours, h (IQR)	15.5 (15.0 − 16.1)	15.2 (14.6 − 15.7)
Compositional mean of sleep, SED, LPA and MVPA, min	497, 584, 282, 77	520, 572, 272, 76

*BMI* Body mass index, *IQR* interquartile range

^a^Included 4 (2%) those with underweight before retirement and 5 (2%) after retirement.

Among the current study population smoking was less common (4% vs. 9%), and mobility limitations were rarer (8% vs. 14%) compared to the survey-only study population. Moreover, the current study population had lower self-reported BMI (26.0 vs. 26.8 kg/m^2^) and higher self-reported non-occupational physical activity (26.9 vs. 23.4 MET-hours/week) (Supplement 3).

**Fig. 1:**
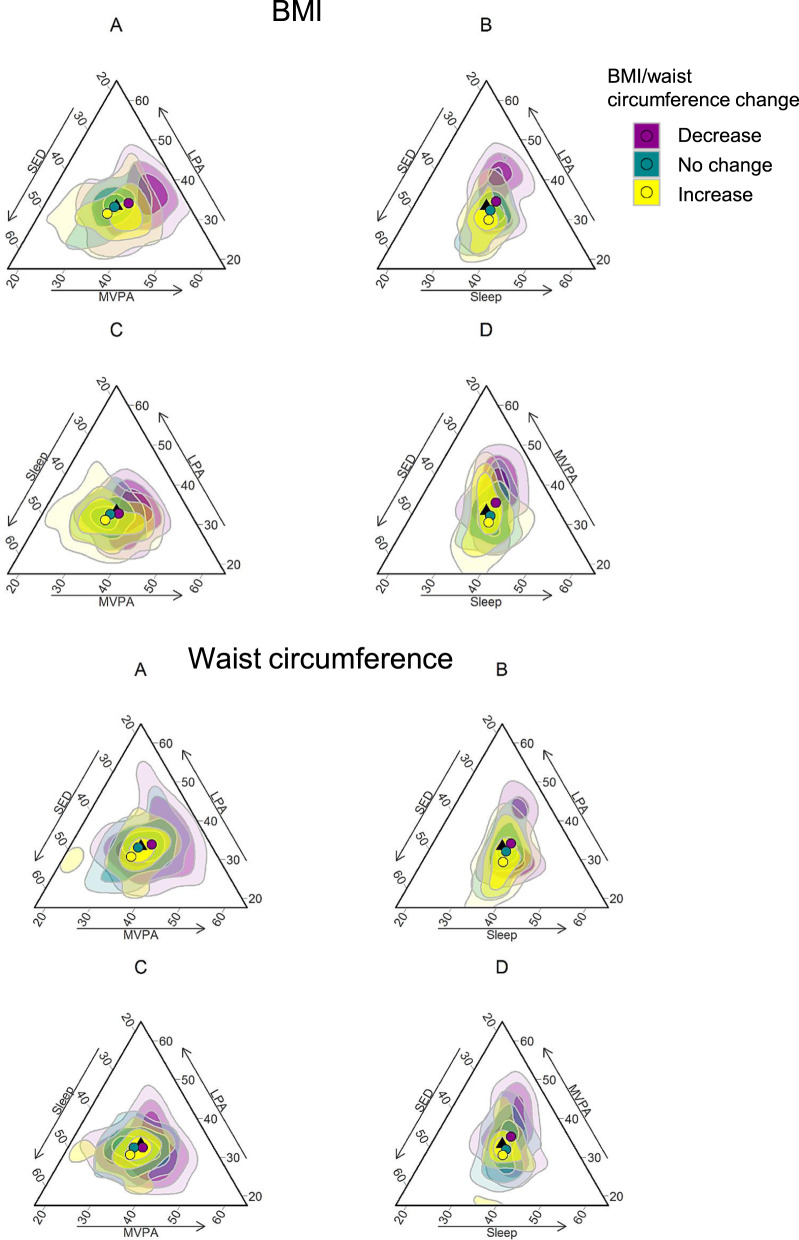
Changes in the 24-h movement behavior composition and changes in BMI and waist circumference. The changes in the 24-h movement behavior composition are visualized as ternary plots in the three-dimensional sub-compositions (**A**, **B**, **C**, and **D**). Black triangle indicates the center of the ternary plot that is, no change in the proportions between 24-h movement behaviors. Colored dots indicate the mean changes in the 24-h movement behaviors and colored areas indicate observations, that is, changes in the proportions between 24-h movement behaviors by obesity indicator change groups. Purple color represents decreasing BMI/waist circumference, turquoise color no changes in BMI/waist circumference and yellow color increasing BMI/waist circumference. The density of the color represents the number of individuals; lighter color indicates low density and darker color higher density. MVPA moderate-to-vigorous physical activity, LPA light physical activity, SED sedentary time.

Changes in the 24-h movement behavior composition according to changes in BMI and waist circumference after the retirement transition are illustrated in Fig. 1. As seen in ternary plots B and D, on average the proportion of sleep and SED increased in relative to both LPA and MVPA among those whose BMI and waist circumference increased (yellow dot and area). In contrast, the proportion of LPA and MVPA increased related to sleep and SED among those whose BMI and waist circumference decreased (plots B and D, purple dot and area). No marked changes in the 24-h movement behavior composition were observed among those whose BMI and waist circumference did not change.

Table 2 presents the longitudinal associations between the one-year changes in 24-h movement behaviors and the changes in BMI and waist circumference from work to retirement. Increasing MVPA in relation to the remaining behaviors was associated with decreasing BMI (β_ilr_ = −0.60, *p* = 0.04) and waist circumference (β_ilr_ = −2.14, *p* = 0.05). Increasing LPA in relation to the remaining behaviors had no statistically significant associations with changes in BMI (β_ilr_ = −0.66, *p* = 0.06) or waist circumference (β_ilr_ = −1.76, *p* = 0.17). Increasing sleep was associated with an increase in BMI (β_ilr_ = 1.34, *p* = 0.02), but not with waist circumference (β_ilr_ = 1.51, *p* = 0.48). Increasing SED in relation to the remaining behaviors was not associated with changes in BMI (β_ilr_ = −0.09, *p* = 0.85), or waist circumference (β_ilr_ = 2.39, *p* = 0.15). Additional adjustments for the before retirement measurement season and follow-up time did not notably change the results (Supplement 4). When long sleepers (over 9 h) were excluded, associations between increasing sleep and BMI attenuated, whereas association between increasing SED and increasing waist circumference became statistically significant (Supplement 5).

**Table 2:** Associations between changes in 24-h movement behaviors (expressed as ilr coordinates, only the first pivot coordinate presented) and changes in BMI and waist circumference.

	Body Mass Index (kg/m^2^)		Waist circumference (cm)	
	β_ilr_ (95% CI)	*p* value	β_ilr_ (95% CI)	*p* value
Sleep vs remaining, difference	1.34 (0.21 to 2.47)	0.02	1.51 (−2.71 to 5.73)	0.48
SED vs remaining, difference	−0.09 (−0.96 to 0.78)	0.85	2.39 (−0.86 to 5.65)	0.15
LPA vs remaining, difference	−0.66 (−1.33 to 0.02)	0.06	−1.76 (−4.30 to 0.77)	0.17
MVPA vs remaining, difference	−0.60 (−1.16 to −0.04)	0.04	−2.14 (−4.25 to −0.03)	0.05

Adjusted for baseline body mass index (BMI)/waist circumference, baseline 24-h movement behavior composition, age, sex and occupation.

**Fig. 2:**
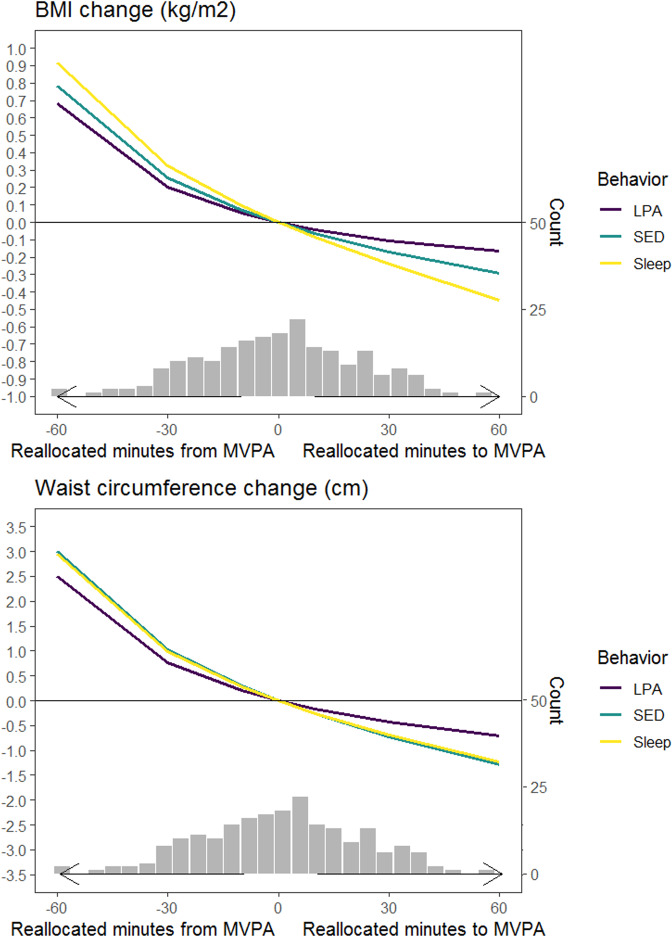
One-to-one reallocations between MVPA and sleep, MVPA and SED, MVPA and LPA and changes in BMI and waist circumference over one-year from work to retirement. Histograms show the distribution of real changes observed in MVPA in the study population.

Figure 2 and Supplement 6 show how the one-to-one reallocations between MVPA and sleep, MVPA and SED, MVPA and LPA were associated with the changes in BMI and waist circumference. Reallocation of time from MVPA to sleep and from MVPA to SED increased BMI and waist circumference in a relatively similar manner: BMI by +0.78 − 0.91 kg/m^2^ and waist circumference by +3.0 cm with a 60-min reallocation, by +0.25 − 0.32 kg/m^2^ and +1.0 cm with a 30-min reallocation, and by +0.07 − 0.09 kg/m^2^ and +0.3 cm with a 10-min reallocation. Reallocation of time from MVPA to LPA was also associated with an increased BMI and waist circumference, but to a smaller extent (Supplement 6). Overall, the effects of increasing MVPA were smaller compared with the effects of decreasing MVPA (Fig. 2). Also, reallocating time between sleep, SED and LPA were associated with markedly smaller changes in BMI and waist circumference than those between MVPA and sleep, SED and LPA (Supplement 7). For instance, a 90-min increase in LPA at the cost of SED was needed to observe similar changes in BMI and waist circumference when compared to increasing 30 min of MVPA at the cost of SED.

## Discussion

This natural experiment study, over a one-year period in the transition from work to retirement, showed that increasing MVPA in relation to the remaining 24-h movement behaviors was associated with decreasing BMI and waist circumference. In contrast, increasing sleep was associated with increasing BMI. Thus, changes in MVPA and sleep had contrasting effects on these obesity indicators, which is important and novel knowledge in relation to public health recommendations for people transitioning into retirement.

Our findings support the well-known health benefits of MVPA in preventing obesity [4]. However, the novelty of our study is that we were able to show how the change in obesity indicators depended on whether or which of the 24-h movement behaviors MVPA replaced or was replaced with. We observed that the beneficial effect of increasing MVPA did not markedly differ when MVPA replaced sleep or SED, but slightly smaller benefits were observed when MVPA replaced LPA. Given that MVPA is located at the high end of the energy expenditure continuum [49], replacing behaviors located at the low end of the continuum (sleep, SED) with MVPA increases the overall energy expenditure. This may lead to weight loss and decrease in body fat if the energy intake remains constant. In line with previous studies [23, 50, 51], the effect of decreasing MVPA was larger compared with the effect of increasing MVPA, highlighting the importance of maintaining MVPA levels when retiring to prevent weight gain and central obesity.

Epidemiological evidence suggests a U-shaped curve between sleep duration and health, i.e., it is detrimental to sleep too little but also too much [7–9]. Our study complements the knowledge by showing that increasing sleep at the cost of physical activity was associated with slight increases in BMI and waist circumference. This may be explained by decreased energy expenditure. Unfortunately, these findings concern a majority of the retirees, as sleep is shown to increase after retirement [24, 28, 40], e.g., in our study population by 23 min and the increase in sleep mainly contributes to decreasing LPA and MVPA among retirees [28]. However, associations between changes in sleep in relation to the remaining 24-h movement behaviors and obesity indicators may depend on the baseline sleep duration levels. For example, increasing sleep duration from insufficient level generally improves cardiometabolic health and reduces adiposity [52–54]. In the current study, the majority of the study participants had already recommended level of sleep (7–9 h per night) [55] before the retirement transition. It is therefore possible that increasing sleep time from an already sufficient level only decreased overall energy expenditure without providing any additional benefits for metabolism that are typically associated with adequate sleep [14, 56]. Given that our study sample included very few individuals with short (14%) or long (12%) sleep, future studies with larger proportion of short and long sleepers are needed to elucidate how the baseline sleep duration affects these associations.

Previous cross-sectional studies have shown beneficial associations between higher proportion of sleep in relation to SED and obesity indicators [19–21]. In our study, we did not observe notable benefits for BMI and waist circumference when SED was replaced with sleep. Both sleep and SED have very low energy expenditure, which may explain why reallocations between them did not markedly change these obesity indicators. Moreover, it should be noted that since sleep time was based on self-reported waking and bedtimes and we did not have information on sleep efficiency, we cannot be certain of how much of the increased sleep time represents actual sleep versus how much of it consists of lying in bed awake, i.e., sedentary time. Thus, the possible beneficial effects of sleep may be slightly underestimated when using self-reported sleep time.

Our findings give indications of how retirees should spend their time in 24-h movement behaviors to prevent weight gain and central obesity. Given that the detrimental effect of decreasing MVPA was larger compared with beneficial effect of increasing MVPA, we conclude that maintaining MVPA levels after retirement is important. This may also be a more feasible goal than aiming to increase MVPA levels, because the pre-retirement MVPA levels may be relatively high (in this study population about 80 min/day). Moreover, a large proportion of pre-retirement daily physical activity accrues from worktime physical activity, which may be difficult to compensate after retirement, especially for those with physically demanding occupations [25, 57]. On the other hand, among people who actively commute by foot or bike, regular physical activity is accrued during workdays [25]. It has been shown that those who engage in active commuting before retirement tend to maintain their activity level relatively well after retirement [25]. We observed some benefits of replacing SED with LPA, but compared to that of MVPA a threefold increase in LPA was needed to gain approximately same amount of reduction in BMI and waist circumference. This is in line with findings from the previous longitudinal study applying the CoDA methodology [23]. Our findings also suggest that increasing sleep when retiring may not be as detrimental for health in terms of obesity, if it does not decrease daily total physical activity. However, because studies are showing the opposite, compensation of the removed physical activities due to retirement can be achieved by engaging in regular leisure time MVPA such as organized activities, brisk walking and cycling, but also in active lifestyle throughout the day for instance by breaking up sedentary time at home, doing heavy household chores and active commuting.

We observed associations between changes in 24-h movement behaviors and BMI and waist circumference over one year, but larger effects could be expected with longer follow-up periods. For instance, in our study reallocating 30 min from MVPA to SED was estimated to increase BMI by 0.25 kg/m^2^, whereas in the 7-year follow-up study among Central European older women (mean baseline age 63.9) [23] the effect on BMI was estimated to be 0.75 kg/m^2^. However, it should be noted that sleep was not included in the estimations of that study [23], and thus, the effect of MVPA may be overestimated. Therefore, future studies covering changes in all 24-h movement behaviors over longer follow-up periods are needed.

The most important strengths of this study is a longitudinal study design, which decreases the risk of self-selection and the effect of time-invariant confounders [58, 59]. Using repeated accelerometer-based measurements of 24-h movement behaviors instead of self-reports, the risk of recall and information bias is removed [60]. We used posture-based identification of SED, LPA and MVPA with ActiPASS (Acti4) which has shown to identify postures and physical activities with high sensitivity (80%) and specificity (>90%) during semi-standardized and free-living conditions [33, 35]. In addition, we used appropriate statistical methods to examine changes and reallocations between codependent components of a 24-h day [47]. We also took into account the seasonal effects on the findings by conducting measurements at the same time of the year and adjusting the analyses for measurement season and follow-up length.

Our study naturally has some limitations. We examined concurrent short-term changes in 24-h movement behaviors and BMI and waist circumference, thus we were unable to show the direction of causation. Our study sample was relatively small, which increases the risk of type II errors (failure to identify significant effect that actually exists) [61]. We did not have information on changes in body composition, which could also have changed due to reallocation of the movement behaviors. Moreover, we were unable to separate contexts of physical activity (for instance physically strenuous work tasks, strength training). These aspects may be relevant, because removal of physically strenuous work tasks may reduce muscle mass and body weight, whereas opposite changes may occur if strength training increases after retirement. In addition to changes in movement behaviors, there may also be other factors affecting changes in obesity indicators that we did not measure, such as energy intake. Previous studies have reported for example decreasing consumption of vegetables and increasing consumption of fruits after retirement [62]. Aging is associated with loss of muscle mass and increase in total body fat [63] but age-related changes unlikely affected our results notably due to the short follow-up period. Sleep estimates were based on self-reported measures which reflect time in bed rather than actual sleep time. However, there were no marked differences in sleep estimates between participant log used in the current study and accelerometer-based sleep detection methods, which are considered as more reliable methods to estimate sleep in field-based studies [64]. Finally, given that the current study population consisted mainly of women (82%) and non-manual workers (69%) and was generally leaner, healthier, and more active compared with the survey-only study participants, the generalizability of the findings may be limited.

In conclusion, increasing MVPA in relation to sleep, SED and LPA over one year during transition from work to retirement was associated with decreasing BMI and waist circumference, whereas increasing sleep was associated with increasing BMI. Future studies are needed to elucidate the long-term effects of changes in 24-h movement behaviors on BMI, waist circumference and body composition.

### Supplementary information

Supplement 1

Supplement 2

Supplement 3

Supplement 4

Supplement 5

Supplement 6

Supplement 7

## Data Availability

Anonymised partial datasets of the FIREA study are available by application with bona fide researchers with an established scientific record and bona fide organisations. In case of data requests, please contact the principal investigator Sari Stenholm, sari.stenholm@utu.fi.
