# Supplementary material for: Modifications of 24-h movement behaviors to prevent obesity in retirement: a natural experiment using compositional data analysis
Source: Int J Obes (Lond). 2023 May 23;47(10):922–30. doi: 10.1038/s41366-023-01326-0 (PMC10511314; doi:10.1038/s41366-023-01326-0)
Supplement: Supplementary file 1 — Supplement 1 [file 41366_2023_1326_MOESM1_ESM.docx]

# **Supplement 1.docx.** Flow chart for the selection of the study population.

Excluded:

Already retired/sick leave n=7

Did not participate in accelerometer and clinical measurements before and after retirement n=40

Device malfunction n=3

Excluded:

Had less than 3 valid measurement days before and/or after retirement n=27

Final analytical sample n=213

Provided accelerometer and obesity indicator data both before and after retirement n=240

Clinical sub-study participants n=290

**Finnish Retirement and Aging study (FIREA)** n=6783
