# Supplementary material for: Modifications of 24-h movement behaviors to prevent obesity in retirement: a natural experiment using compositional data analysis
Source: Int J Obes (Lond). 2023 May 23;47(10):922–30. doi: 10.1038/s41366-023-01326-0 (PMC10511314; doi:10.1038/s41366-023-01326-0)
Supplement: Supplementary file 2 — Supplement 2 [file 41366_2023_1326_MOESM2_ESM.docx]

# **Supplement 2.docx. Statistical analyses**

*Descriptive analysis of compositional data*

The compositional means were calculated as the component-wise geometric means of the data, and rescaled to sum up to 1440 min. The compositional differences between pre- and post-retirement compositions were calculated for each participant via perturbation, which is a compositional operation analogous to addition or subtraction^1^. In practice, this meant scaling each observation so that the sum of its compositional parts was one [1], and then dividing each part of the post-retirement observation with the corresponding part of the pre-retirement observation. The resulting composition of the compositional differences by obesity indicator change groups was visualized as ternary plots.

*Changing compositional data to real space*

Following the CoDA principles, compositional data was transformed to isometric logratios (*ilr*), which reduces the dimensionality of the data, allows compositional data to be expressed in real space and enables compositional parts to be used as such in standard statistical models^2,3^. We used *pivot coordinates,* a specific type of *ilr* coordinates^4^. Since we had a four-part composition (sleep, SED, LPA, MVPA) we created four sets of pivot coordinates:

1. $z_{1\_sleep}= \sqrt{\frac{3}{4}}\ln\frac{Sleep}{\sqrt[3]{SED x LPA x MVPA}} , z_{2\_sleep}= \sqrt{\frac{2}{3}}\ln\frac{SED}{\sqrt[2]{LPA x. MVPA}} , z_{3\_sleep}= \sqrt{\frac{1}{2}}ln\frac{LPA}{\sqrt[1]{MVPA}}$

2. $z_{1\_SED}= \sqrt{\frac{3}{4}}\ln\frac{SED}{\sqrt[3]{Sleep x LPA x MVPA}} , z_{2\_SED}= \sqrt{\frac{2}{3}}\ln\frac{Sleep}{\sqrt[2]{LPA x. MVPA}} , z_{3\_SED}= \sqrt{\frac{1}{2}}ln\frac{LPA}{\sqrt[1]{MVPA}}$

3. $z_{1\_LPA}= \sqrt{\frac{3}{4}}\ln\frac{LPA}{\sqrt[3]{Sleep x SED x MVPA}} , z_{2\_LPA}= \sqrt{\frac{2}{3}}\ln\frac{Sleep}{\sqrt[2]{SED x MVPA}} , z_{3\_LPA}= \sqrt{\frac{1}{2}}ln\frac{SED}{\sqrt[1]{MVPA}}$

4. $z_{1\_MVPA}= \sqrt{\frac{3}{4}}\ln\frac{MVPA}{\sqrt[3]{Sleep x SED x LPA}} , z_{2\_MVPA}= \sqrt{\frac{2}{3}}\ln\frac{Sleep}{\sqrt[2]{SED x LPA}} , z_{3\_MVPA}= \sqrt{\frac{1}{2}}ln\frac{SED}{\sqrt[1]{LPA}}$

One set of pivot coordinates (e.g. z1_sleep, z2_sleep, z3_sleep) represents the four-part composition and lower case (_sleep, _SED, _LPA & _MVPA) indicates which compositional part is set at the first position in the composition. When a specific compositional part is set at the first position, it means that all relative information about this compositional part is contained in the first coordinate^4^. Pivot coordinates are therefore useful when specifically modeling associations between changes in a single part in a composition (i.e. sleep/SED/LPA/MVPA) in relation to the remaining behaviors and changes in health indicators.

*Linear regression models*

To examine how changes in the 24-h movement behavior composition were associated with changes in BMI and waist circumference, we used following linear regression model:

$\Delta Obesity indicator=\beta_{0}+\beta_{1}^{\left( MB1 \right)}z_{1}^{\left( MB1 \right)}+ {\beta_{2}^{\left( MB1 \right)}z}_{2}^{\left( MB1 \right)}+ {\beta_{3}^{\left( MB1 \right)}z}_{3}^{\left( MB1 \right)}+ \beta_{4}^{\left( MB\triangle\right)}z_{1}^{\left( MB\triangle\right)}+ \beta_{5}^{\left( MB\triangle\right)}z_{2}^{\left( MB\triangle\right)}+{\beta_{6}^{\left( MB\triangle\right)}z_{3}^{\left( MB\triangle\right)}+ \beta}_{7}Obesity indicator1+\beta_{8}Age1+\beta_{9}Sex+\beta_{10}Occupation+\varepsilon,$

- $Obesity indicator1, \Delta Obesity indicator$: before retirement obesity indicator, i.e. BMI/Waist circumference, change in obesity indicator during the transition to retirement
- $Age1$: before retirement age
- $Sex:$man/woman
- $Occupation$: manual/non-manual occupation before retirement
- $z_{1}^{\left( MB1 \right)}, z_{2}^{(MB1)}$, $z_{3}^{(MB1)}$: pivot coordinates for the before retirement 24-h movement behavior composition, $MB$ in the upper index indicates which compositional part is set at the first position in the composition
- $z_{1}^{\left( MB\triangle\right)}, z_{2}^{(MB\triangle)},z_{3}^{(MB\triangle)}$: difference between pivot coordinates for after and before retirement MB composition
- $\varepsilon$… random error term

The linear regression model was repeated for each set of pivot coordinates to allow for each compositional part to be examined in relation to the remaining behaviors.

*Compositional isotemporal substitution*

To illustrate the effect of observed reallocations between 24-h movement behaviors on BMI and waist circumference the compositional isotemporal substitution model was used^5,6^. For this purpose, we used previously described linear regression model, where MVPA was set as the first compositional part in the pivot coordinate set.

As the baseline composition, we used the mean before retirement composition, closed to 1440 min (24 hours) per day (sleep 497 min, SED 584 min, LPA 282 min, MVPA 77 min). The follow-up composition was obtained by taking the baseline composition, subtracting x minutes from a certain compositional part and adding those x minutes to another compositional part, while keeping rest of the compositional parts constant. These systematic reallocations between movement behaviors were calculated to reflect the “measured changes” in the composition. We chose the sizes of these reallocations (x minutes) based on the actual measured range of change from baseline to follow-up in 24-h movement behaviors. Consequently, one-to-one reallocation between MVPA and one of the remaining behaviors (sleep/SED/LPA) were up to 60 minutes in size, while one-to-one reallocations between sleep, SED and LPA were up to 120 minutes in size. Thereafter, each reallocated composition was transformed into pivot coordinates, and the difference between the coordinates of each reallocated composition and the baseline composition was calculated. After that, the regression based coefficients were applied on the calculated differences in order to predict how changes in obesity indicator correspond to changes in composition of movement behaviors during the transition from work to retirement. The model-estimated standard error of the change in obesity indicators was used to derive 95% confidence intervals (CI) for the estimated change in obesity indicators. The distribution of actual measured changes in 24-h movement behaviors was illustrated as histograms.

References

1. Pasanen J, Leskinen T, Suorsa K, Pulakka A, Virta J, Auranen K *et al.* Effects of physical activity intervention on 24-h movement behaviors: a compositional data analysis. *Sci Rep* 2022; 12. doi:10.1038/s41598-022-12715-2.

2. Aitchison J. The Statistical Analysis of Compositional Data. *J R Stat Soc Ser B Methodol* 1982; 44: 139–177.

3. Pawlowsky-Glahn V, Egozcue J, Tolosana-Delgado R. Modelling and analysis of compositional data. John Wiley & Sons: Chichester, UK, 2015.

4. Hron K, Filzmoser P, de Caritat P, Fišerová E, Gardlo A. Weighted Pivot Coordinates for Compositional Data and Their Application to Geochemical Mapping. *Math Geosci* 2017; 49: 797–814.

5. Dumuid D, Pedišić Ž, Stanford T, Martín-Fernández J, Hron K, Maher C *et al.* The compositional isotemporal substitution model: A method for estimating changes in a health outcome for reallocation of time between sleep, physical activity and sedentary behaviour. *Stat Methods Med Res* 2019; 28: 846–857.

6. Olds T, Burton N, Sprod J, Maher C, Ferrar K, Brown W *et al*. One day you’ll wake up and won’t have to go to work: The impact of changes in time use on mental health following retirement. *PloS One* 2018; **13**. doi:10.1371/journal.pone.0199605.
