# Supplementary material for: Modifications of 24-h movement behaviors to prevent obesity in retirement: a natural experiment using compositional data analysis
Source: Int J Obes (Lond). 2023 May 23;47(10):922–30. doi: 10.1038/s41366-023-01326-0 (PMC10511314; doi:10.1038/s41366-023-01326-0)
Supplement: Supplementary file 3 — Supplement 3 [file 41366_2023_1326_MOESM3_ESM.docx]

**Supplement 3.docx.** Comparison of the preretirement characteristics between the study population included in the analyses (n=213) and the survey-only study population (n=3698) in the last available measurement in which the participants were still working.

| Characteristics | Study population  n=213 | Survey-only study population  n=3698 | p value |
| --- | --- | --- | --- |
| Age, mean (SD) | 63.5 (1.1) | 63.4 (1.4) | 0.76 |
| Women, n (%) | 175 (82) | 3056 (83) | 0.86 |
| Occupational group, n (%) |  |  | 0.19 |
| Manual | 67 (31) | 1314 (36) |  |
| Non-manual | 146 (69) | 2350 (64) |  |
| Current smoking, n (%) | 9 (4) | 301 (9) | 0.02 |
| Chronic diseases, n (%) | 134 (67) | 2175 (67) | 0.87 |
| Mobility limitation, n (%) | 17 (8) | 470 (14) | 0.02 |
| Body Mass Index, mean (SD), kg/m^2a^ | 26.0 (4.2) | 26.8 (4.5) | 0.01 |
| Self-reported sleep duration, h, mean (SD) | 7.2 (0.86) | 7.1 (0.87) | 0.84 |
| Self-reported daily total sitting time, h, mean (SD) | 8.2 (2.7) | 8.0 (3.1) | 0.40 |
| Self-reported non-occupational physical activity, MET-hours, mean (SD) | 26.9 (18.9) | 23.4 (19.7) | 0.01 |

^a^Based on self-reported height and weight.
