# Supplementary material for: Modifications of 24-h movement behaviors to prevent obesity in retirement: a natural experiment using compositional data analysis
Source: Int J Obes (Lond). 2023 May 23;47(10):922–30. doi: 10.1038/s41366-023-01326-0 (PMC10511314; doi:10.1038/s41366-023-01326-0)
Supplement: Supplementary file 4 — Supplement 4 [file 41366_2023_1326_MOESM4_ESM.docx]

**Supplement 4.docx.** Associations between changes in 24-h movement behaviors (expressed as ilr coordinates, only the first pivot coordinate presented) and changes in BMI and waist circumference by taking into account measurement season.

|  | Body Mass Index (kg/m^2^) | | Waist circumference (cm) | |
| --- | --- | --- | --- | --- |
|  | β_ilr_ (95% CI) | p value | β_ilr_ (95% CI) | p value |
| Sleep vs remaining, difference | 1.22 (0.08 to 2.36) | 0.04 | 1.35 (-2.92 to 5.62) | 0.53 |
| SED vs remaining, difference | 0.00 (-0.88 to 0.88) | 0.99 | 2.57 (-0.73 to 5.87) | 0.13 |
| LPA vs remaining, difference | -0.61 (-1.29 to 0.07) | 0.08 | -1.77 (-4.32 to 0.78) | 0.17 |
| MVPA vs remaining, difference | -0.61 (-1.17 to -0.05) | 0.03 | -2.15 (-4.27 to -0.03) | 0.05 |

Adjusted for baseline body mass index (BMI)/waist circumference, baseline 24-h movement behavior composition, age, sex, occupation, before retirement measurement season (winter/ spring/summer/autumn) and follow-up time in days.
