# Supplementary material for: Modifications of 24-h movement behaviors to prevent obesity in retirement: a natural experiment using compositional data analysis
Source: Int J Obes (Lond). 2023 May 23;47(10):922–30. doi: 10.1038/s41366-023-01326-0 (PMC10511314; doi:10.1038/s41366-023-01326-0)
Supplement: Supplementary file 5 — Supplement 5 [file 41366_2023_1326_MOESM5_ESM.docx]

**Supplement 5.docx.** Associations between changes in 24-h movement behaviors (expressed as ilr coordinates, only the first pivot coordinate presented) and changes in BMI and waist circumference among the study population without long sleepers (n=187).

|  | Body Mass Index (kg/m^2^) | | Waist circumference (cm) | |
| --- | --- | --- | --- | --- |
|  | β_ilr_ (95% CI) | p value | β_ilr_ (95% CI) | p value |
| Sleep vs remaining, difference | 1.10 (-0.06 to 2.25) | 0.06 | -0.14 (-4.46 to 4.19) | 0.95 |
| SED vs remaining, difference | 0.19 (-0.69 to 1.06) | 0.67 | 3.51 (0.23 to 6.79) | 0.04 |
| LPA vs remaining, difference | -0.66 (-1.37 to 0.05) | 0.07 | -0.94 (-3.61 to 1.72) | 0.49 |
| MVPA vs remaining, difference | -0.63 (-1.19 to -0.07) | 0.03 | -2.43 (-4.54 to -0.31) | 0.03 |

Adjusted for baseline body mass index (BMI)/waist circumference, baseline 24-h movement behavior composition, age, sex and occupation.
