# Supplementary material for: Modifications of 24-h movement behaviors to prevent obesity in retirement: a natural experiment using compositional data analysis
Source: Int J Obes (Lond). 2023 May 23;47(10):922–30. doi: 10.1038/s41366-023-01326-0 (PMC10511314; doi:10.1038/s41366-023-01326-0)
Supplement: Supplementary file 6 — Supplement 6 [file 41366_2023_1326_MOESM6_ESM.docx]

**Supplement 6.docx.** One-to-one reallocations between MVPA and sleep, MVPA and SED, MVPA and LPA and changes in BMI and waist circumference and their 95% confidence intervals (CI).

| Reallocation | Body Mass Index change (kg/m^2^)  Mean (95% CI) | |  | Waist circumference change (cm)  Mean (95% CI) | |  |
| --- | --- | --- | --- | --- | --- | --- |
|  | 10 min | 30 min | 60 min | 10 min | 30 min | 60 min |
| MVPA to sleep | 0.09 (0.07 to 0.12) | 0.32 (0.27 to 0.37) | 0.91 (0.61 to 1.22) | 0.28 (0.21 to 0.36) | 0.99 (0.81 to 1.18) | 2.95 (1.79 to 4.11) |
| MVPA to SED | 0.07 (0.05 to 0.09) | 0.25 (0.20 to 0.31) | 0.78 (0.45 to 1.11) | 0.29 (0.21 to 0.38) | 1.01 (0.80 to 1.23) | 2.99 (1.75 to 4.23) |
| MVPA to LPA | 0.05 (0.03 to 0.08) | 0.20 (0.14 to 0.27) | 0.68 (0.31 to 1.05) | 0.20 (0.11 to 0.29) | 0.75 (0.51 to 1.00) | 2.49 (1.12 to 3.86) |
| Sleep to MVPA | -0.09 (-0.11 to -0.07) | -0.24 (-0.28 to -0.21) | -0.45 (-0.51 to -0.38) | -0.25 (-0.33 to -0.18) | -0.69 (-0.83 to -0.56) | -1.24 (-1.51 to -0.98) |
| SED to MVPA | -0.06 (-0.08 to -0.04) | -0.17 (-0.20 to -0.14) | -0.29 (-0.35 to -0.24) | -0.26 (-0.33 to -0.19) | -0.72 (-0.82 to -0.61) | -1.29 (-1.51 to -1.07) |
| LPA to MVPA | -0.04 (-0.06 to -0.02) | -0.11 (-0.14 to -0.08) | -0.17 (-0.25 to -0.09) | -0.17 (-0.24 to -0.10) | -0.43 (-0.56 to -0.31) | -0.69 (-0.99 to -0.39) |
