# Supplementary material for: Modifications of 24-h movement behaviors to prevent obesity in retirement: a natural experiment using compositional data analysis
Source: Int J Obes (Lond). 2023 May 23;47(10):922–30. doi: 10.1038/s41366-023-01326-0 (PMC10511314; doi:10.1038/s41366-023-01326-0)

**Supplement 7. docx.** One-to-one reallocations between sleep and SED, sleep and LPA, LPA and SED and changes in BMI and waist circumference and their 95% confidence intervals (CI) over one-year from work to retirement. Histograms show the distribution of real changes observed in sleep/LPA in the study population.


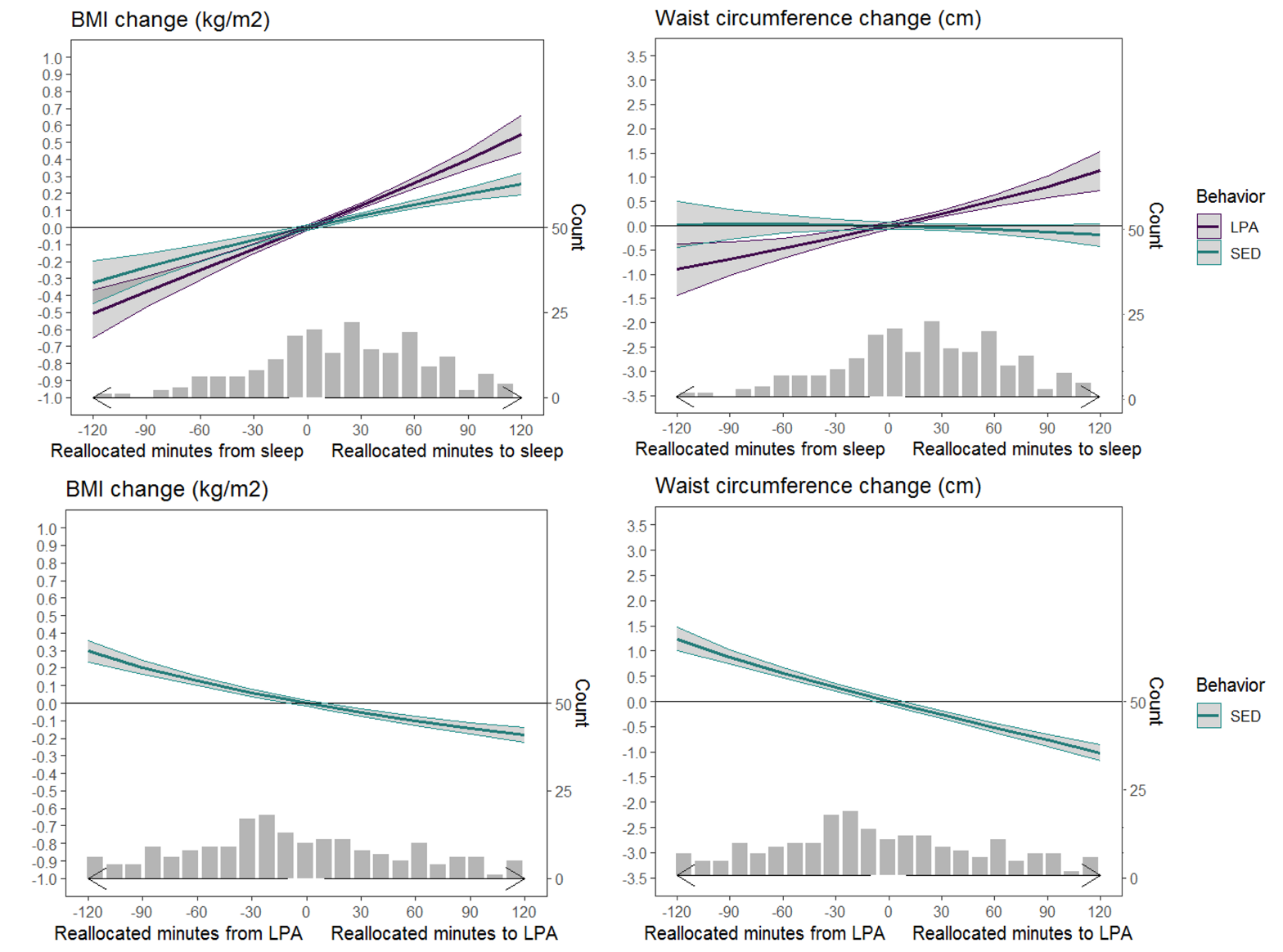

Supplement: Supplementary file 7 — Supplement 7 [file 41366_2023_1326_MOESM7_ESM.docx]
